# 15 Years Out: Reinventing ICCVAM

**DOI:** 10.1289/ehp.1206292

**Published:** 2013-02-01

**Authors:** Linda S. Birnbaum

**Affiliations:** Director, NIEHS and NTP, National Institutes of Health, Department of Health and Human Services, Research Triangle Park, North Carolina, E-mail: birnbaumls@niehs.nih.gov

In 1997, the National Institute of Environmental Health Sciences (NIEHS) established the Interagency Coordinating Committee on the Validation of Alternative Methods (ICCVAM), an ad hoc federal interagency committee to address the growing need for obtaining regulatory acceptance of new toxicological test methods. The thought was that simultaneous agency evaluation of new methods that addressed the 3Rs (reduction, refinement, and replacement) of animal testing by an interagency group could greatly speed up and harmonize the cross-agency acceptance and adoption of new methods into federal toxicity testing guidelines. This activity was codified into law in 2000 by passage of the ICCVAM Authorization Act (2000). The Act specified 15 agencies (such as the Food and Drug Administration, U.S. Environmental Protection Agency, Consumer Product Safety Commission, Department of Transportation, Occupational Safety and Health Administration, and U.S. Department of Agriculture) that would constitute ICCVAM. The Act also prescribed specific duties intended to facilitate review and acceptance of test methods, established an external scientific advisory committee, and required the director of the NIEHS to establish ICCVAM under the National Toxicology Program (NTP) Interagency Center for the Evaluation of Alternative Toxicological Methods (NICEATM), which currently exists as a functional unit within the Division of the NTP at the NIEHS.

Over the past 15 years, ICCVAM has successfully evaluated and recommended numerous alternative test methods for regulatory use (NTP 2012). However, the lack of implementation of ICCVAM-recommended methods has been an area of increasing concern. The NIEHS has worked proactively with our ICCVAM partners to identify promising methods, encouraged and aided test developers in building a case for validating their methods, sometimes provided financial support through competitive Small Business Innovation Grants, and held workshops and engaged our federal and international partners to promote acceptance and use of test methods in specific areas of toxicology (e.g., ocular toxicity and skin sensitization). Even so, regulatory use of alternative methods has still lagged behind. Critics have repeatedly pointed out that alternative test methods have not been accepted for regulatory decision making and that the expectations for real reductions in animal use in toxicology testing have always outpaced the documented progress. It has become clear that it is time to change our approach.

The NIEHS is beginning to move forward with a different philosophy toward ICCVAM. Rather than the NIEHS directing the activities of ICCVAM through NICEATM, the interagency agenda will now be driven by the partner regulatory agencies—the agencies that will ultimately implement the ICCVAM-recommended methods. Regulatory agencies are required by statute to use toxicology test information for a variety of purposes, including labeling and registration, and these requirements are not uniform. The ICCVAM Authorization Act acknowledges that some alternative test methods promoted by ICCVAM, while deemed valid, may not meet specific needs of a regulatory agency. With ICCVAM regulatory agencies taking ownership of the process, there should be a better match between the alternative test methods validated and the tests required to meet regulatory guidelines.

Toxicology testing is shifting from a primary focus on adverse phenotypic observations in animals to mechanism-based biological outcomes *in vitro*, and the NIEHS is embracing this paradigm shift through its participation in the multiagency Tox21 consortium (Collins et al. 2008). NICEATM will expand its scope and concentrate its resources on providing bioinformatic and computational toxicology support to NIEHS Tox21 projects.

With its purpose of transforming toxicology by shifting from *in vivo* animal studies to *in vitro* assays, *in vivo* assays in lower organisms, and computational modeling for toxicity assessments, Tox21 has the real potential to result in dramatic changes in the numbers and types of organisms used for toxicology testing. A stronger interface of NICEATM with Tox21 will better position ICCVAM for addressing how data from these new methods can be integrated into the existing regulatory framework.

We express our deep appreciation to William S. Stokes, who has served as the director of NICEATM since its inception. In December 2012, he retired from the Public Health Service after 33 years of dedicated federal service. His vision, persistence, and direction have been key to bringing NICEATM, ICCVAM, and the International Cooperation on Alternative Test Methods (ICATM) to their current stage of maturity.

We are pleased that Warren Casey, who has served as deputy director of NICEATM, will now serve as the acting director. He is uniquely qualified for this role, having worked in the areas of toxicogenomics, mechanistic toxicology, and biomarker development in the pharmaceutical industry prior to joining the NIEHS.

We look forward to this new approach to promoting the 3Rs—an approach that will be driven by regulatory agency needs while remaining responsive to the test method development community.
